# Hepatic compartmentalization of exhausted and regulatory cells in HIV/HCV-coinfected patients

**DOI:** 10.1111/jvh.12291

**Published:** 2014-09-01

**Authors:** L. Barrett, N. Trehanpati, S. Poonia, L. Daigh, S. Kumar Sarin, H. Masur, S. Kottilil

**Affiliations:** 1 Laboratory of Immunoregulation, NIAID, National Institutes of Health, Bethesda, MD, USA; 2 Institute of Liver and Biliary Sciences, New Delhi, India

**Keywords:** HCV, HIV, intrahepatic immunity, T cell

## Abstract

Accelerated intrahepatic hepatitis *C* virus (HCV) pathogenesis is likely the result of dysregulation within both the innate and adaptive immune compartments, but the exact contribution of peripheral blood and liver lymphocyte subsets remains unclear. Prolonged activation and expansion of immunoregulatory cells have been thought to play a role. We determined immune cell subset frequency in contemporaneous liver and peripheral blood samples from chronic HCV-infected and HIV/HCV-coinfected individuals. Peripheral blood mononuclear cells (PBMC) and biopsy-derived liver-infiltrating lymphocytes from 26 HIV/HCV-coinfected , 10 chronic HCV-infected and 10 HIV-infected individuals were assessed for various subsets of T and B lymphocytes, dendritic cell, natural killer (NK) cell and NK T-cell frequency by flow cytometry. CD8^+^ T cells expressing the exhaustion marker PD-1 were increased in HCV-infected individuals compared with uninfected individuals (*P* = 0.02), and HIV coinfection enhanced this effect (*P* = 0.005). In the liver, regulatory CD4^+^CD25^+^Foxp3^+^ T cells, as well as CD4^+^CD25^+^PD1^+^ T cells, were more frequent in HIV/HCV-coinfected than in HCV-monoinfected samples (*P* < 0.001). HCV was associated with increased regulatory T cells, PD-1^+^ T cells and decreased memory B cells, regardless of HIV infection (*P* ≤ 0.005 for all). Low CD8^+^ expression was observed only in PD-1^+^CD8^+^ T cells from HCV-infected individuals and healthy controls (*P* = 0.002) and was associated with enhanced expansion of exhausted CD8^+^ T cells when exposed *in vitro* to PHA or CMV peptides. In conclusion, in HIV/HCV coinfection, ongoing HCV replication is associated with increased regulatory and exhausted T cells in the periphery and liver that may impact control of HCV. Simultaneous characterization of liver and peripheral blood highlights the disproportionate intrahepatic compartmentalization of immunoregulatory T cells, which may contribute to establishment of chronicity and hepatic fibrogenesis in HIV coinfection.

## INTRODUCTION

Hepatitis *C* virus (HCV) infection is an ongoing epidemic, with approximately 150 million people infected worldwide [1]. Once infected with HCV, most individuals develop a lifelong infection that results in liver-related morbidity and mortality, including cirrhosis, decompensated liver disease requiring transplant and hepatocellular carcinoma [2]. Coinfection with HIV, another bloodborne pathogen, is common due to shared routes of transmission. HIV coinfection accelerates the course of liver disease, with faster progression to clinically significant hepatic fibrosis [3]. However, the course of HIV-associated disease (CD4^+^ T-cell count, HIV viral load) remains largely unaffected during HCV coinfection [4].

HCV maintains a productive infection over many years in otherwise immunocompetent individuals via various immune escape mechanisms [5]. In the context of overt immunosuppression, HCV-related liver disease often progresses from indolent to fulminant, suggesting an ongoing role for the immune system in limiting HCV pathogenesis [6].

Development of progressive liver fibrosis and eventually cirrhosis is accompanied by concomitant loss of function. Whether this stems directly from HCV replication or is the result of collateral damage from long-standing inflammation is unclear [7]. Liver-infiltrating lymphocytes (LIL) are thought to represent the inflammatory response to HCV and play a role in the pathogenesis of chronic hepatitis *C*. Previous studies reported increased exhausted and regulatory T cells in both the peripheral blood and the liver [8,9] and an association between functional intrahepatic IFN-gamma-producing cells and lower fibrosis scores [10]. However, other studies suggest immunoregulatory, and not IFN-gamma-producing cells, are associated with less intrahepatic fibrosis [11]. The status of intrahepatic lymphocytes in HIV/HCV-coinfected individuals and the roles they play in accelerated hepatic fibrogenesis are largely unknown. It is conceivable that these cells provide valuable insights into the pathogenesis of chronic inflammation and fibrosis.

We comprehensively studied the peripheral blood and intrahepatic immunophenotype of adaptive and innate cells in HIV/HCV-coinfected and HCV-monoinfected populations to assess quantitative and qualitative differences characteristic of the altered intrahepatic immunologic constitution that may explain the clinical course of HIV/HCV coinfection.

## METHODS

### Study subjects

HIV/HCV- and HCV-infected patients were recruited between July 2008 and November 2011 to a prospective study of the natural history of HCV monoinfection and HIV/HCV coinfection at the National Institute of Allergy and Infectious Diseases (NIAID). Patients were at least 18 years of age, not receiving HCV therapy, HCV positive by HCV RNA assay (Roche Amplicor, Roche Molecular Diagnostics, Pleasanton, CA, USA) and HCV seropositive, and coinfected patients were also HIV positive by HIV assay (Roche Amplicor, Roche Molecular Diagnostics, Pleasanton, CA, USA) and EIA. All patients provided written informed consent for PBMC collection and storage, and protocol indicated liver biopsy.

Healthy volunteers were recruited through an ongoing study of healthy volunteers at the NIAID. Patients were at least 18 years of age, and HIV, HCV and HBV negative. All patients provided written informed consent for PBMC collection and storage. Both studies were reviewed and approved by the NIAID Institutional Review Board, with annual renewal.

### Peripheral blood mononuclear cell (PBMC) and liver-infiltrating lymphocyte (LIL) isolation

Twenty-six HIV/HCV-coinfected, 10 HCV-monoinfected, and 10 HCV- and HIV-negative individuals underwent two-pass leukapheresis or peripheral venipuncture. PBMC were isolated by Ficoll-Hypaque (Life Technologies, Grand Island, NY, USA) and density gradient centrifugation, as previously described [12]. Cells were counted by trypan blue exclusion and stored in liquid nitrogen at a centralized repository until use.

LIL were isolated from the liver tissue biopsy samples via mechanical dissociation. Patients underwent ultrasound-guided percutaneous liver biopsy, and an 18-gauge biopsy core was immediately placed in 5-mL lymphocyte medium (RPMI supplemented with 10% foetal calf serum, 1% penicillin and streptomycin, 1 mm HEPES and 20 micromolar of 2-mercaptoethanol [all from Life technologies]) and was processed within 30 min of collection. The liver core was washed with Hank’s solution containing 2% FCS and 1% EDTA to remove peripheral blood. Following the wash, the tissue was whittled into small pieces and homogenized according to manufacturer’s instructions using a Medimachine (BD Biosciences, San Jose, CA, USA). The resulting cell suspension was passed through a 70-μm cell strainer and centrifuged. The upper part of suspension was carefully recovered, layered on Ficoll-Hypaque separation solution, and LIL were isolated by density gradient centrifugation. The viability of isolated cells was determined by trypan blue exclusion. In general, >1 × 10^6^ LIL were obtained from 1 g of liver tissue, and viable LIL were >90%.

### Immunophenotyping

Peripheral blood mononuclear cells were phenotyped in HIV/HCV-coinfected (*n* = 26), HCV-monoinfected (*n* = 10), and HIV-, HCV-, and HBV-uninfected (*n* = 10) patients. Multicolour flow cytometry analyses were performed on thawed PBMCs that had been stored in liquid nitrogen. The following fluorochrome-conjugated monoclonal antibodies were used for T-cell phenotyping: APC-Cy7 anti-CD3, FITC anti-CD4, Horizon V500 anti-CD8, PE anti-CD27, PE-Cy7 anti-CD28, eFluor450 anti-CD57 (eBioscience, San Diego, CA, USA), PerCPCy5.5 anti-PD-1 (Biolegend, San Diego, CA, USA), APC anti-Tim-3 (eBioscience), FITC anti-CCR7 (Biolegend), FITC anti-CD2 and PerCPCy5.5 anti-Foxp3. All antibodies are from BD Biosciences (San Jose, CA, USA) unless otherwise noted. T regulatory, dendritic cell, monocyte and NK cell phenotyping were performed at the NIAID core facility using the following antibodies (BD Biosciences, San Jose, CA, USA): CD3, CD38, HLA-DR, CD45RO, CD19, CD25, Foxp3, CD27, CD56, CD16, BDCA2, CD123, CD209 (DC-SIGN) and CD14. Analyses were performed on a FACS CantoII flow cytometer (BD Biosciences) with FlowJo version 8.6 software (TreeStar, Ashland, OR, USA).

### Cell stimulation

To determine the effect of HIV coinfection on T-cell response to stimulation, 2.5 × 10^6^ PBMC from CMV-positive, HIV/HCV-coinfected and HCV-monoinfected individuals were incubated at 37 °C, 5% CO_2_ for 18 h with either 5 μg/mL phytohaemagglutinin (PHA; Sigma-Aldrich, St. Louis MO), 2.5 μg/mL human cytomegalovirus (CMV) pp65 15-mer peptides (Miltenyi Biotec, Auburn, CA, USA), 2.5 μg/mL HCV 15-mer peptides (BEI Resources) or lymphocyte medium. Cells were harvested and immunophenotyped using the T-cell panels described above.

### Statistical analysis

Comparisons of independent groups were made by the Wilcoxon two-sample test.

The Wilcoxon signed rank test was used to compare paired data. The Bonferroni method was used to adjust *P* values for multiple testing. Analyses were conducted using SPSS version 19.0 (IBM Corporation, Armonk, New York) and GraphPad Prism version 5.03 (GraphPad Prism Software Inc., San Diego, CA, USA).

## RESULTS

### Demographics

The study was composed of 36 HCV-positive patients, whose clinical characteristics are shown in Table 1. Of this study population, 10 (28%) were HCV-monoinfected patients and 26 (72%) were HIV/HCV-coinfected patients. The majority of the HIV-coinfected cohort was male, while the HCV-monoinfected cohort was predominantly female (Male 73% *vs* 30%, *P* = 0.03) with higher HCV viral loads (*P* = 0.006). The two cohorts were similar with regards to age, race and HCV genotype. HIV disease was well controlled in the coinfected cohort (mean CD4 counts 719 cells/mm^3^ and HIV VL <40 copies/mL). Both cohorts exhibited mild liver disease. One HCV-monoinfected patient did not undergo a liver biopsy.

### In peripheral blood, HCV and HIV/HCV patients have distinct immunophenotypes

**Fig. 1:**
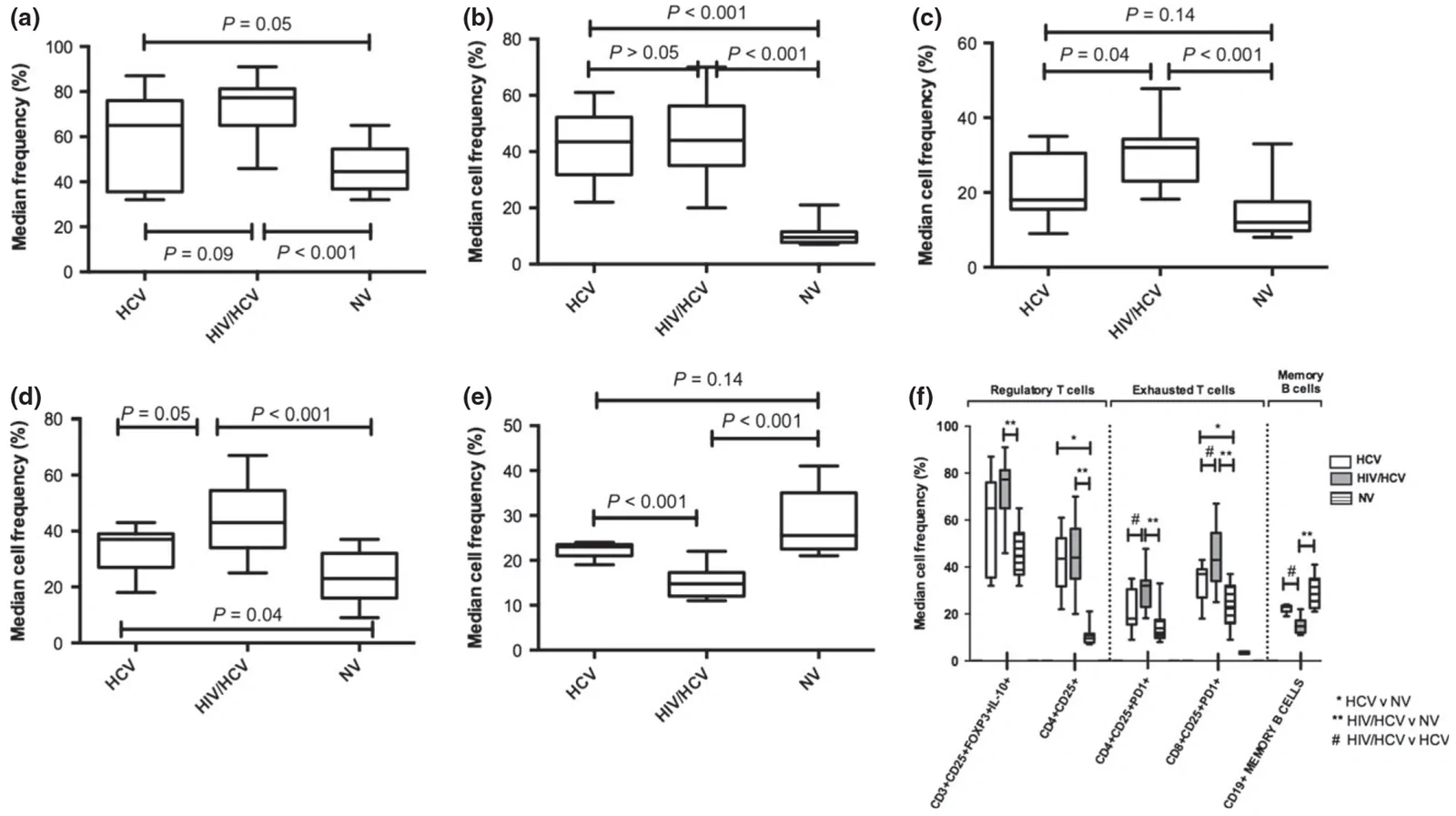
Peripheral blood immune phenotype in HCV monoinfection and HIV/HCV coinfection. Cryopreserved PBMC were thawed and stained immediately for surface markers. Data are expressed as median frequency and interquartile range. HIV/HCV-coinfected (HIV/HCV) *n* = 26, HCV-monoinfected (HCV) *n* = 10, uninfected (NV) *n* = 10. (a) Frequency of regulatory T cells expressing IL-10 in peripheral blood. (b) Frequency of CD4^+^CD25^+^ T cells in peripheral blood. (c) Frequency of CD4^+^CD25^+^PD-1^+^ T cells in peripheral blood. (d) Frequency of CD8^+^CD25^+^PD-1^+^ T cells in peripheral blood. (e) Frequency of memory B cells in peripheral blood. (f) Summary of significant changes in peripheral blood with HCV infection. Wilcoxson’s two-sample test was used to compare groups. * – statistically significant difference between HCV-monoinfected group and uninfected volunteers. ** – statistically significant difference between HIV/HCV-coinfected group and uninfected volunteers. # – statistically significant difference between HIV/HCV-coinfected group and HCV-monoinfected group.

Peripheral blood mononuclear cells (PBMC) were enumerated by flow cytometry in HCV-monoinfected (*n* = 10), HIV/HCV-coinfected (*n* = 26) and uninfected (*n* = 10) study participants. HCV infection was associated with an increased frequency of CD3^+^CD25^+^Foxp3^+^ regulatory T cells expressing IL-10, which was further increased in the context of HIV coinfection (Fig. 1a). Moreover, the frequency of CD4^+^CD25^+^ regulatory T cells was significantly higher in both HCV-monoinfected individuals (Fig. 1b; 43.5 [34, 53]) and HIV/HCV-coinfected individuals (44.0 (33.5, 54.5) compared with normal volunteers (9.5 (8.1, 10.9); *P* < 0.001).

There was a significantly higher frequency of CD4^+^CD25^+^PD1^+^ cells in the peripheral blood of HIV/HCV-coinfected individuals (Fig. 1c; 32.0 [21, 43]) than in HCV-monoinfected individuals (18.0 (13, 23); *P* = 0.04) and normal volunteers (12.0 (9,15)]; *P* < 0.001). Similarly, the frequency of CD8^+^CD25^+^PD1^+^ T cells was higher in HIV/HCV-coinfected individuals (Fig. 1d; 43.0 [33, 53]) compared with HCV-monoinfected individuals (37.0 (33.5, 40.5); *P* = 0.05) and normal volunteers (23.0 (16, 30); *P* < 0.001). Furthermore, HIV coinfection was associated with a decreased frequency of CD19^+^ memory B cells (Fig. 1e; (14.8 (12.3, 17.3); *P* < 0.001) relative to HCV-monoinfected individuals (23.0 [22, 24]) and normal volunteers (25.5, [20.1, 30.9]).

### Regulatory T cells are significantly increased in the liver of HIV/HCV-coinfected individuals compared with HCV-monoinfected individuals

**Fig. 2:**
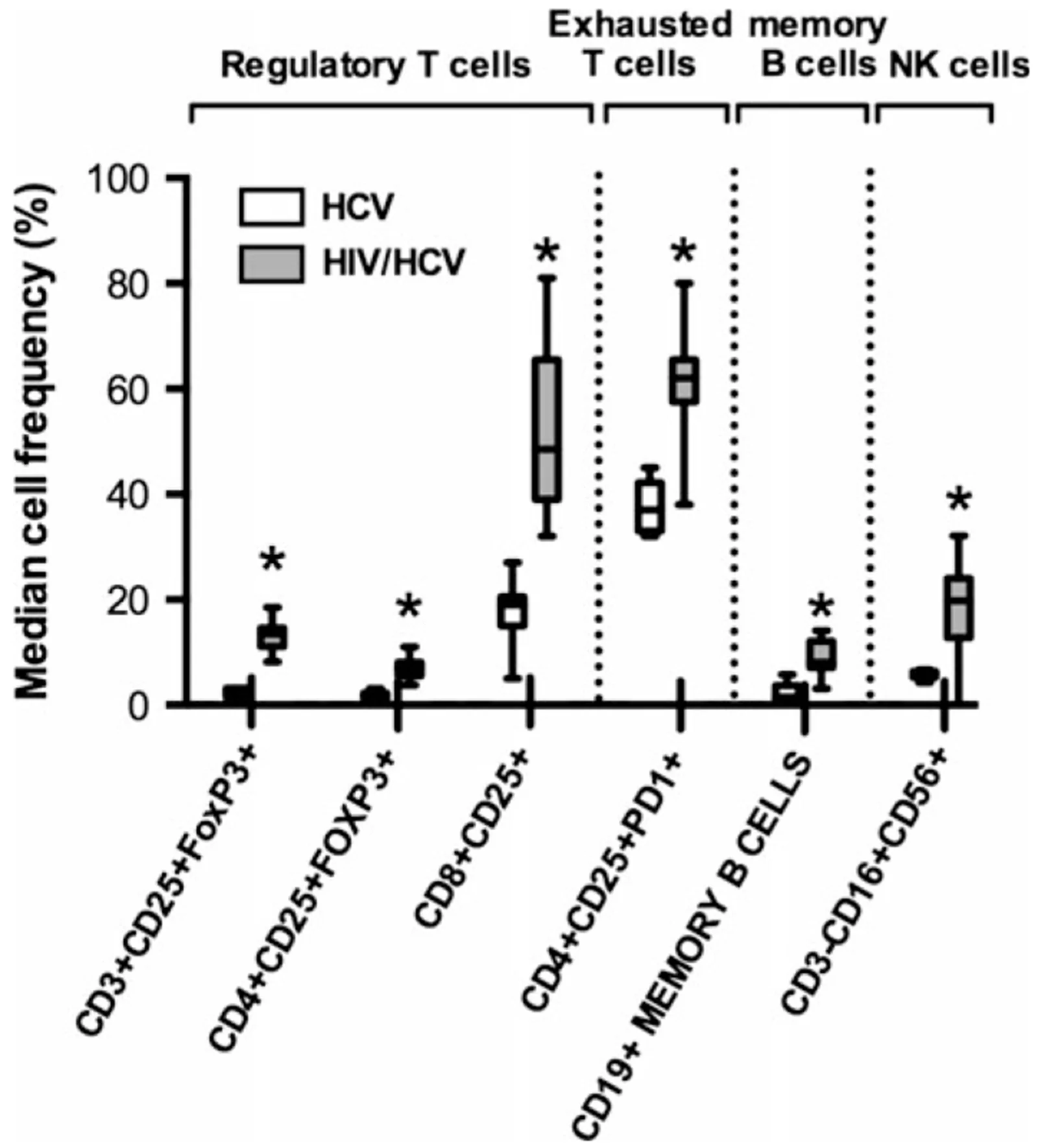
Liver-infiltrating lymphocyte immune phenotype in HCV monoinfection and HIV/HCV coinfection. Percutaneous liver biopsies were immediately processed to isolate liver-infiltrating lymphocytes and stained for the described markers. Data are expressed as median frequency and interquartile range. HIV/HCV-coinfected (HIV/HCV) *n* = 26 and HCV-monoinfected (HCV) *n* = 9. Wilcoxson’s two-sample test was used to compare groups. * – statistically significant difference between HIV/HCV-coinfected and HCV-monoinfected group.

The liver normally exists as an immunoregulatory environment. However, the effect of HIV infection on the intrahepatic environment is unclear, particularly in the context of chronic hepatitis due to HCV infection. We compared the frequency of various intrahepatic regulatory T cells in HCV-monoinfected and HIV/HCV-coinfected individuals. Several regulatory T-cell populations were increased in HIV/HCV-coinfected patients relative to HCV-monoinfected patients (Fig. 2): CD3^+^CD25^+^Foxp3^+^ T cells (13.45 (11.13, 14.63) *vs* 2.4 (1.45, 3.2); *P* = 0.006); CD4^+^CD25^+^Foxp3^+^ T cells (7.1, (5.85, 8.35) *vs* 1.7 (1.4, 2.0); *P* = 0.00003); and CD8^+^CD25^+^ T cells (48.5 (35.5, 61.5) *vs* 19.0 (18,20); *P* = 0.00004). Moreover, the frequency of CD4^+^CD25^+^PD1^+^ exhausted T cells was significantly higher in HIV/HCV-coinfected individuals compared with HCV-monoinfected individuals (62.0 (58.5, 65.5) *vs* 37.0 (33.5, 40.5); *P* = 0.000003).

Intrahepatic CD19^+^ memory B cells were more frequent in HIV/HCV-coinfected individuals than in HCV-monoinfected individuals (8.0 (5.5, 10.5) *vs* 1.5 (0.75, 2.25); *P* = 0.0000002). CD3^−^CD16^+^CD56^+^ NK cell frequencies were significantly higher in the HIV/HCV-coinfected group compared with the HCV-monoinfected group (19.8 (14.0, 25.4) *vs* 5.6 (5.2, 6.0); *P* = 0.000014).

### Increased frequencies of regulatory and exhausted cells are present in the liver compared with the peripheral blood during HCV infection

**Fig. 3:**
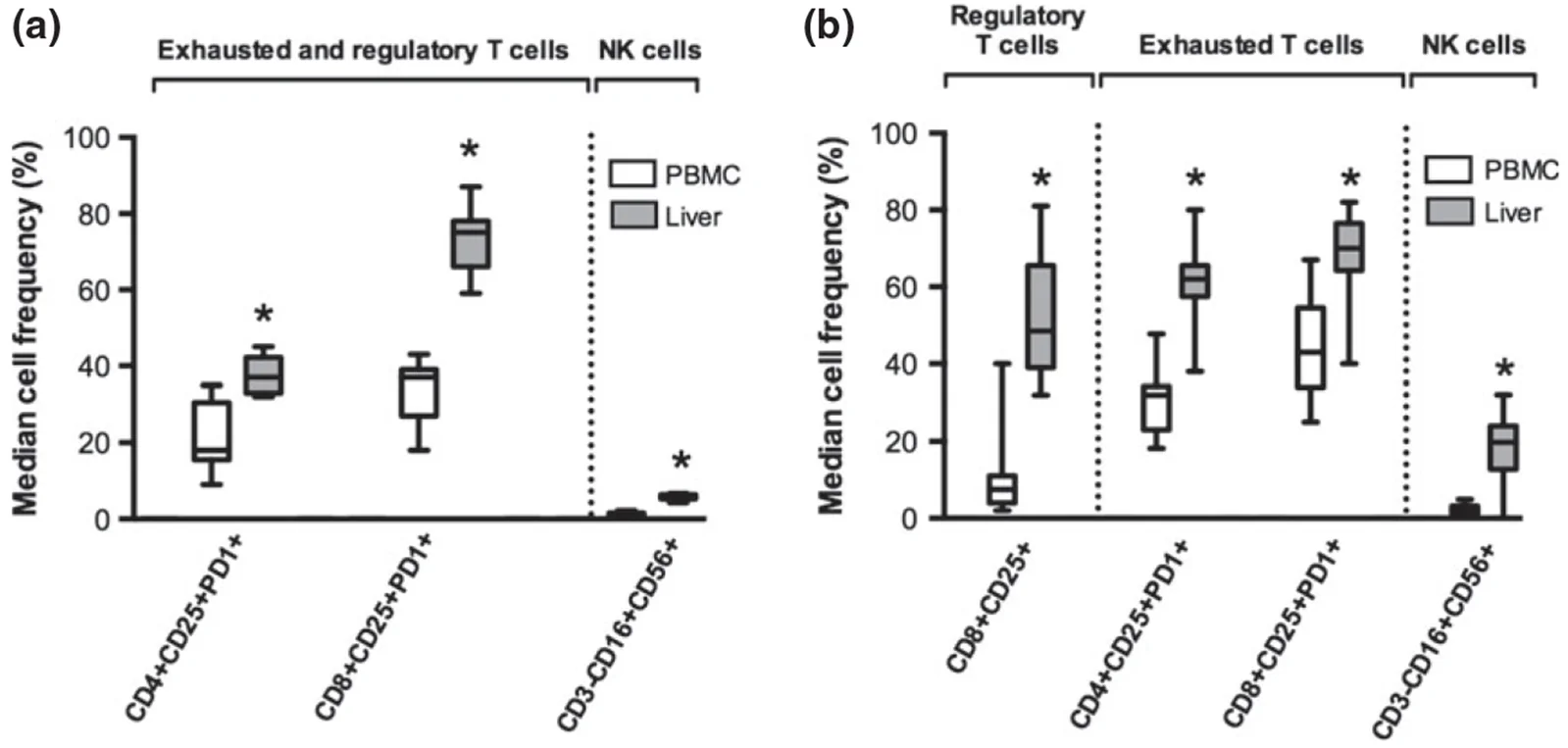
Comparison of immune cells in the liver and periphery. Data are expressed as median frequency and interquartile range. HIV/HCV-coinfected (HIV/HCV) *n* = 26 and HCV-monoinfected (HCV) *n* = 9. Wilcoxson’s two-sample test was used to compare groups. (a) Regulatory, exhausted and NK cell frequency in liver and peripheral blood in HCV monoinfection, *n* = 9. (b) Regulatory, exhausted and NK cell frequency in liver and peripheral blood in HIV/HCV coinfection, *n* = 26. * – statistically significant difference between HIV/HCV-coinfected and HCV-monoinfected group

We next compared the frequency of various immune cell subsets in the liver and peripheral blood. There was no significant difference in Foxp3^+^ regulatory CD4^+^ or CD8^+^ T cells, NKT cells or plasmacytoid dendritic cells (data not shown). In both the HCV-monoinfected group and the HIV/HCV-coinfected group, the frequency of CD25^+^ PD1^+^ exhausted, regulatory T cells was significantly higher in the liver than in the peripheral blood (Fig. 3). NK cells were also significantly higher in the liver compared with the periphery in both groups. The CD8^+^CD25^+^ regulatory T-cell frequency in the HIV/HCV-coinfected group was significantly higher in the liver compared with the periphery (Fig. 3b; 48.5 (39.0, 65.5) *vs* 7.5 (4,11); *P* = 0.001), while no such differences were observed in the HCV-monoinfected group.

### Impaired CD8 expression and high PD-1 expression with stimulation characterize CD8^+^ T cells in HCV infection

**Fig. 4:**
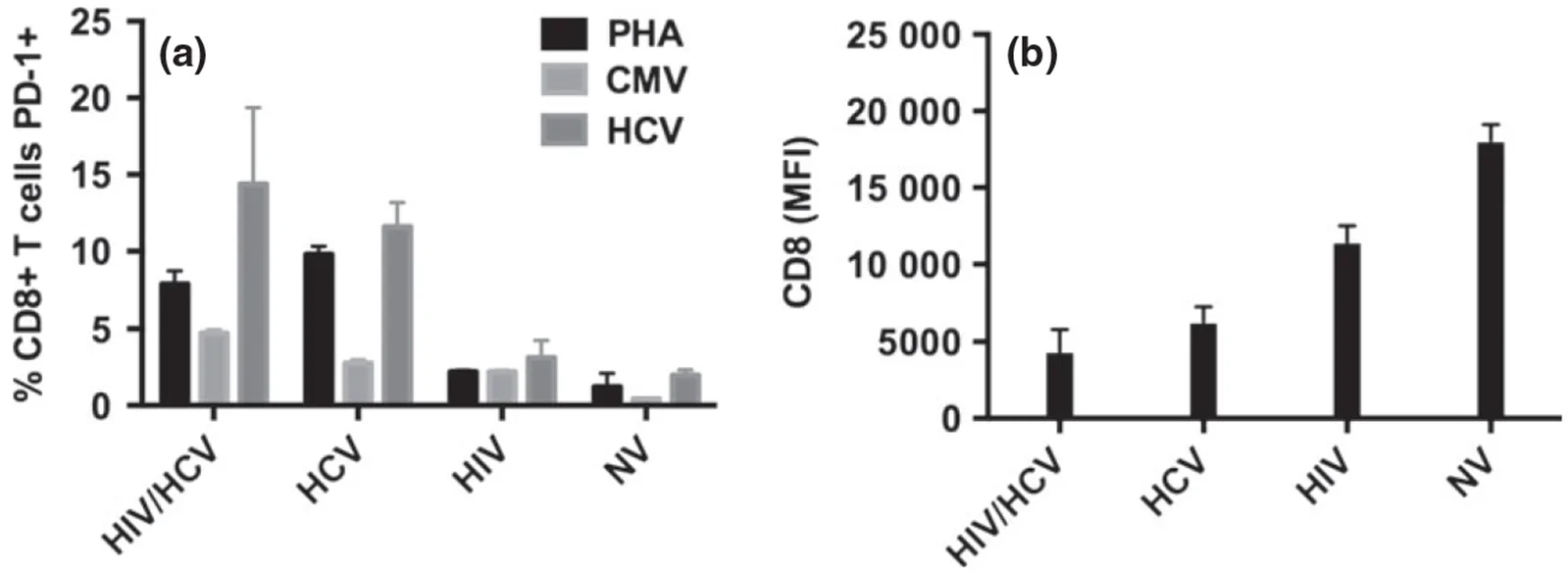
T-cell response to stimulation in HIV/HCV coinfection, HCV monoinfection and HIV infection. PBMC were incubated for 18 h with mitogen (PHA), HCV, or CMV and immune phenotype was assessed by flow cytometry. HIV/HCV *n* = 10, HCV *n* = 10 and uninfected (NV) *n* = 10. Student’s *t*-test was used to compare groups. (a) Expression of the exhaustion marker PD-1 on CD8^+^ T cells. Data are expressed as mean frequency and standard deviation. (b) CD8 expression with PHA stimulation. Data are expressed as mean frequency and standard deviation. MFI – mean fluorescence intensity *P < 0.05

Given the high frequency of exhausted and regulatory T cells observed in the HCV-infected population, particularly the HIV/HCV-coinfected group, we assessed whether *in vitro* T-cell response to mitogen and recall antigen would be altered. We noted no significant changes in CD4^+^ T-cell and NK cell responses between the HIV/HCV (*n* = 5), HIV (*n* = 5), HCV (*n* = 5), and uninfected (*n* = 5) PBMC (data not shown). Stimulation with mitogen or HCV peptides resulted in an increased frequency of CD8^+^PD-1^+^ T cells in the HCV-monoinfected and HIV/HCV-coinfected groups compared with HIV-infected and uninfected groups (Fig. 4a). Moreover, decreased expression of the essential T-cell costimulatory molecule CD8 was observed in the PBMCs from HIV/HCV-, HCV- and HIV-infected subjects upon mitogenic stimulation (Fig. 4b). This effect was most pronounced in the PBMCs from HCV-infected subjects (both HIV/HCV coinfected and HCV monoinfected). The effect of CMV peptide stimulation was more modest; in all groups, less than 5 per cent of CD8^+^ T cells were PD1^+^ upon stimulation. The frequency of PD-1^+^ CD8 T cells did not differ with the type of stimulation in HCV-uninfected groups (Fig. 4a).

Overall, these results demonstrate that stimulation of T cells with either global T-cell mitogen or recall antigen increases exhaustion markers in CD8^+^ T cells from HCV-infected individuals regardless of HIV infection. Furthermore, HCV peptide stimulation is a more potent inducer of PD-1 expression than other stimuli.

## DISCUSSION

Our results demonstrate that HIV coinfection of HCV-infected individuals leads to a distinct immunophenotype, both in the liver and periphery, characterized by enrichment of exhausted and regulatory T cells compared with HCV-monoinfected subjects. Moreover, exhausted and regulatory T cells were more prevalent in the liver than in the periphery of all HCV-infected individuals, regardless of HIV serostatus. HCV-specific expansion of CD8 + T cells *in vitro* resulted in an exhausted phenotype characterized by high expression of PD-1 and decreased CD8 expression, while CMV-, HIV- and PHA-stimulated CD8+ T cells did not. These results highlight two important aspects of HCV immunology: (i) the significance of studying intrahepatic T cells and (ii) the influence of HIV coinfection on impaired HCV immunity and accelerated fibrogenesis.

Prior studies of smaller cohorts [13,14] suggested that HCV infection increases the frequency of regulatory and exhausted T cells in the liver and the periphery. In this study, we went a step further and compared the immune phenotype and function in the liver and periphery of HCV-infected patients with or without HIV coinfection. We found that HIV coinfection exacerbated the exhausted phenotype, particularly in the liver, where CD25^+^PD-1^+^ exhausted T cells were significantly more prevalent than in the periphery. Previous studies have correlated PD-1 expression with HCV disease severity in HIV/HCV-coinfected populations [12]. Antibody blockade of the interaction between PD-1 and its ligands has been shown to restore functional capabilities in exhausted HCV- and HBV-specific T cells *in vitro* [13,15] and lead to decreased HCV viremia in a chimpanzee model of HCV [16]. Taken together, these results suggest that HIV coinfection leads to an immunological milieu that predisposes to the development of virologic persistence and the more rapid progression to advanced liver disease observed in HIV-infected chronic HCV patients.

Furthermore, we found that HCV infection alone altered the frequency of PD-1^+^ CD8^+^ T cells upon *in vitro* stimulation with mitogen or recall antigen when compared to normal volunteers. Chronic HCV infection was associated with an increased frequency of PD-1^+^ CD8^+^ T cells, and decreased CD8 expression upon mitogenic stimulation was observed in PBMCs from both HCV-monoinfected and HIV/HCV-coinfected individuals. Low CD8 expression has been associated with poor CD8^+^ T-cell activation and responsiveness to antigen [17]. Thus, it is clear that continuous antigenic stimulation results in increased frequency of intrahepatic and peripheral blood PD-1^+^ exhausted T cells in chronic HCV patients, which may dampen the ability of the infected host to mount effective antiviral immunity. As both HCV-monoinfected and HIV/HCV-coinfected patients presented with a similar degree of expansion of CD8^+^ T cells following *in vitro* stimulation, the central cause of diminished anti-HCV immune response remains the expansion of exhausted T cells, rather than an independent effect of HIV infection.

Our study also showed that the effects of HCV infection apply to B cells. In this regard, we observed an increased frequency of intrahepatic memory B cells in the coinfected compared with HCV-monoinfected group. However, memory B cells were less frequent in the peripheral blood of coinfected patients. Such a difference between intrahepatic and peripheral blood frequencies likely reflects preferential compartmentalization of cells in the liver, due to either enhanced homing to the infection site or increased retention upon arrival. Mizuochi *et al*. previously showed high expression of the chemokine receptor CXCR3 on peripheral B cells in chronic hepatitis *C* patients [18]. The chemokine IP-10, a ligand for CXCR3 [19], is an interferon-inducible protein that serves as a marker of hepatic inflammation and is produced at high levels during HCV infection [18]. Moreover, serum levels of IP-10 have been shown to be higher in HIV/HCV-coinfected individuals than in HCV-monoinfected individuals [20]. Therefore, the increased frequency of intrahepatic memory B cells observed here in coinfected patients potentially reflects increased IP-10 production associated with HIV coinfection. In support of this hypothesis, we observed that NK cells, which can also express CXCR3, were increased in liver tissue of the coinfected group. In the context of chronic hepatitis *C*, CXCR3^+^ NK cells have been shown to exhibit impaired degranulation and interferon-gamma production [21]. Therefore, HIV coinfection of chronic hepatitis *C* patients may alter the intrahepatic immune environment in a manner that promotes the development of severe liver disease.

This study was limited by its cross-sectional nature. Longitudinal analysis of peripheral blood and intrahepatic lymphocyte populations in HCV-monoinfected and HIV/HCV-coinfected patients would provide further insight into changes in the immunophenotype observed in these populations and how that relates to the progression of liver disease. We also lack sufficient knowledge of the intrahepatic immune environment in ‘normal’ individuals. Most previous studies of liver-infiltrating lymphocytes in the uninfected individual are in the context of liver transplant or other significant nonviral hepatitis. The activation marker status in truly ‘healthy’ liver is largely unknown.

To our knowledge, the present study is the first to comprehensively characterize the intrahepatic and peripheral blood immunophenotype of HCV-monoinfected and HIV/HCV-coinfected cohorts. We found that HIV infection of chronic HCV patients increased the frequencies of exhausted and regulatory immune cells, particularly in the liver. These results expand our knowledge of the progression of liver disease and may explain how HIV coinfection promotes accelerated liver fibrosis. Future studies are needed to determine the mechanism by which HIV coinfection contributes to immune exhaustion.

## Figures and Tables

**Table 1:** Patient characteristics at study entry

	HCV	HIV/HCV	*P* value
Patients (*n*)	10	26	
Age (y)	50.0 (7.3)	52.2 (5.5)	0.42
Sex (Male, %)	3 (30)	19 (73)	0.03
Race, *n* (% of total HCV infected)			
Black	6 (60)	17 (65.4)	1.00
White	2 (20)	9 (34.6)	0.69
Asian	2 (20)	0	0.07
HCV Genotype, *n* (% of HCV infected)			
1a	6 (60)	15 (57.7)	1.00
1b	3 (30)	5 (19.2)	0.66
Non-1	1 (10)	6 (23.1)	0.65
HCV RNA (log [IU/mL])*	6.2 (0.5)	5.2 (1.6)	0.006
CD4*	874.4 (302.1)	719.3 (363.2)	0.1173
CD4%*	47.4 (8.8)	34.5 (7.9)	0.001
HIV RNA (log [IU/mL])*	–	1.7 (0)	
HIV RNA suppressed, *n*(%)*	–	25 (96.1)	
cART	–	26 (100)	
ALT	65 (38.7)	55.1 (28.3)	0.49
AST	62.8 (48.4)	49.5 (32.1)	0.46
ALP	87.4 (35.8)	103.3 (43.4)	0.29
HAI fibrosis, *n* (%)			
(0–2)	6 (60)	19 (73.1)	0.69
(3–4)	3 (30)	7 (26.9)	0.69
No Liver Bx	1 (10)	0	

* Baseline values expressed as mean (SD).

The bold values indicate statistically significant differences between the two groups.

## Abbreviations:

ALP: Alkaline phosphatase
ALT: Alanine transaminase
AST: Aspartate transaminase
cART: Combined antiretroviral therapy
CMV: Cytomegalovirus
FCS: Foetal calf serum
HAI: Histologic activity index
HCV: Hepatitis *C* virus
HIV: Human immunodeficiency virus
IFN: Interferon
IQR: Interquartile range
LIL: Liver-infiltrating lymphocytes
MFI: Mean fluorescence intensity
NK: Natural killer
PBMC: Peripheral blood mononuclear cells
PD-1: Programmed death 1
PHA: Phytohaemagglutinin
SE: Standard error
TLM: Tissue-like memory B cells
